# Supplementary material for: Early cerebral hypoxia in extremely preterm infants and neurodevelopmental impairment at 2 year of age: A post hoc analysis of the SafeBoosC II trial
Source: PLoS One. 2022 Jan 24;17(1):e0262640. doi: 10.1371/journal.pone.0262640 (PMC8786171; doi:10.1371/journal.pone.0262640)
Supplement: Attachment — Submitted filename: responce to reviewers.docx [file pone.0262640.s001.docx]

Dear Anne Mette Plomgaard,

Thank you for submitting your manuscript to PLOS ONE. After careful consideration, we feel that it has merit but does not fully meet PLOS ONE’s publication criteria as it currently stands. Therefore, we invite you to submit a revised version of the manuscript that addresses the points raised during the review process.


Please submit your revised manuscript by Nov 21 2020 11:59PM. If you will need more time than this to complete your revisions, please reply to this message or contact the journal office at [plosone@plos.org](mailto:plosone@plos.org). When you're ready to submit your revision, log on to [https://www.editorialmanager.com/pone/](https://www.editorialmanager.com/pone/) and select the 'Submissions Needing Revision' folder to locate your manuscript file.

Please include the following items when submitting your revised manuscript:

- A rebuttal letter that responds to each point raised by the academic editor and reviewer(s). You should upload this letter as a separate file labeled 'Response to Reviewers'.
- A marked-up copy of your manuscript that highlights changes made to the original version. You should upload this as a separate file labeled 'Revised Manuscript with Track Changes'.
- An unmarked version of your revised paper without tracked changes. You should upload this as a separate file labeled 'Manuscript'.

If you would like to make changes to your financial disclosure, please include your updated statement in your cover letter. Guidelines for resubmitting your figure files are available below the reviewer comments at the end of this letter.

If applicable, we recommend that you deposit your laboratory protocols in [protocols.io](http://protocols.io/) to enhance the reproducibility of your results. Protocols.io assigns your protocol its own identifier (DOI) so that it can be cited independently in the future. For instructions see: [http://journals.plos.org/plosone/s/submission-guidelines#loc-laboratory-protocols](http://journals.plos.org/plosone/s/submission-guidelines#loc-laboratory-protocols)

We look forward to receiving your revised manuscript.

Kind regards,

Georg M. Schmölzer
Academic Editor
PLOS ONE

Journal Requirements:

When submitting your revision, we need you to address these additional requirements.

1. Please ensure that your manuscript meets PLOS ONE's style requirements, including those for file naming. The PLOS ONE style templates can be found at

[https://journals.plos.org/plosone/s/file?id=wjVg/PLOSOne_formatting_sample_main_body.pdf](https://journals.plos.org/plosone/s/file?id=wjVg/PLOSOne_formatting_sample_main_body.pdf) and

[https://journals.plos.org/plosone/s/file?id=ba62/PLOSOne_formatting_sample_title_authors_affiliations.pdf](https://journals.plos.org/plosone/s/file?id=ba62/PLOSOne_formatting_sample_title_authors_affiliations.pdf)

2. Thank you for including your ethics statement: 'The SafeBoosC II trial was approved by each hospital’s research ethics committee and where required also by the competent authority responsible for medical devices. Written informed parental consent was mandatory before inclusion. The trial was conducted according to the guidelines of the Declaration of Helsinki and the International Conference on Harmonisation good clinical practice.'

a. Please amend your current ethics statement to include the full name of the ethics committee/institutional review board(s) that approved your specific study.

This has been added in the manuscript.

b. Once you have amended this/these statement(s) in the Methods section of the manuscript, please add the same text to the “Ethics Statement” field of the submission form (via “Edit Submission”).
For additional information about PLOS ONE ethical requirements for human subjects research, please refer to [http://journals.plos.org/plosone/s/submission-guidelines#loc-human-subjects-research](http://journals.plos.org/plosone/s/submission-guidelines#loc-human-subjects-research)

 This information is now available.

3. For more information on PLOS ONE's expectations for statistical reporting, please see [https://journals.plos.org/plosone/s/submission-guidelines.#loc-statistical-reporting](https://journals.plos.org/plosone/s/submission-guidelines.#loc-statistical-reporting). Please update your Methods and Results sections accordingly.

4. We noted in your submission details that a portion of your manuscript may have been presented or published elsewhere.

[Yes.
This manuscript contains data previously published. We clearly state in this manuscript that the analyses are conducted this post-hoc -and was not planned per protocol.
I attach related manuscript.]

Please clarify whether this [conference proceeding or publication] was peer-reviewed and formally published. If this work was previously peer-reviewed and published, in the cover letter please provide the reason that this work does not constitute dual publication and should be included in the current manuscript.

 This has been included in the cover letter

5.We note that you have indicated that data from this study are available upon request. PLOS only allows data to be available upon request if there are legal or ethical restrictions on sharing data publicly. For information on unacceptable data access restrictions, please see [http://journals.plos.org/plosone/s/data-availability#loc-unacceptable-data-access-restrictions](http://journals.plos.org/plosone/s/data-availability#loc-unacceptable-data-access-restrictions).

In your revised cover letter, please address the following prompts:

If there are ethical or legal restrictions on sharing a de-identified data set, please explain them in detail (e.g., data contain potentially identifying or sensitive patient information) and who has imposed them (e.g., an ethics committee). Please also provide contact information for a data access committee, ethics committee, or other institutional body to which data requests may be sent.

Following has been included in the cover letter:

This is a small dataset on an easily identified population at named institutions during an identified time period. While we did make the data on the original trial available to researchers, now when the data includes the two-year follow-up, including data on obvious motor disabilities in a subset of children, we find it has greater privacy concerns, and therefore will not share the data.

b) If there are no restrictions, please upload the minimal anonymized data set necessary to replicate your study findings as either Supporting Information files or to a stable, public repository and provide us with the relevant URLs, DOIs, or accession numbers. Please see [http://www.bmj.com/content/340/bmj.c181.long](http://www.bmj.com/content/340/bmj.c181.long) for guidelines on how to de-identify and prepare clinical data for publication. For a list of acceptable repositories, please see [http://journals.plos.org/plosone/s/data-availability#loc-recommended-repositories](http://journals.plos.org/plosone/s/data-availability#loc-recommended-repositories).

We will update your Data Availability statement on your behalf to reflect the information you provide.

6. PLOS requires an ORCID iD for the corresponding author in Editorial Manager on papers submitted after December 6th, 2016. Please ensure that you have an ORCID iD and that it is validated in Editorial Manager. To do this, go to ‘Update my Information’ (in the upper left-hand corner of the main menu), and click on the Fetch/Validate link next to the ORCID field. This will take you to the ORCID site and allow you to create a new iD or authenticate a pre-existing iD in Editorial Manager. Please see the following video for instructions on linking an ORCID iD to your Editorial Manager account: [https://www.youtube.com/watch?v=_xcclfuvtxQ](https://www.youtube.com/watch?v=_xcclfuvtxQ)

I have provided ORCID iD.

7. Please include captions for your Supporting Information files at the end of your manuscript, and update any in-text citations to match accordingly. Please see our Supporting Information guidelines for more information: http://journals.plos.org/plosone/s/supporting-information.

[Note: HTML markup is below. Please do not edit.]

Reviewers' comments:

Reviewer's Responses to Questions

**Comments to the Author**

1. Is the manuscript technically sound, and do the data support the conclusions?

The manuscript must describe a technically sound piece of scientific research with data that supports the conclusions. Experiments must have been conducted rigorously, with appropriate controls, replication, and sample sizes. The conclusions must be drawn appropriately based on the data presented.

Reviewer #1: Partly

Reviewer #2: Partly

2. Has the statistical analysis been performed appropriately and rigorously?

Reviewer #1: Yes

Reviewer #2: No

3. Have the authors made all data underlying the findings in their manuscript fully available?

The [PLOS Data policy](http://www.plosone.org/static/policies.action#sharing) requires authors to make all data underlying the findings described in their manuscript fully available without restriction, with rare exception (please refer to the Data Availability Statement in the manuscript PDF file). The data should be provided as part of the manuscript or its supporting information, or deposited to a public repository. For example, in addition to summary statistics, the data points behind means, medians and variance measures should be available. If there are restrictions on publicly sharing data—e.g. participant privacy or use of data from a third party—those must be specified.

Reviewer #1: Yes

Reviewer #2: No

4. Is the manuscript presented in an intelligible fashion and written in standard English?

PLOS ONE does not copyedit accepted manuscripts, so the language in submitted articles must be clear, correct, and unambiguous. Any typographical or grammatical errors should be corrected at revision, so please note any specific errors here.

Reviewer #1: Yes

Reviewer #2: Yes

5. Review Comments to the Author

Please use the space provided to explain your answers to the questions above. You may also include additional comments for the author, including concerns about dual publication, research ethics, or publication ethics. (Please upload your review as an attachment if it exceeds 20,000 characters)

Reviewer #1: Early cerebral hypoxia in extremely preterm infants and neurodevelopmental impairment at 2 years of age: A post hoc analysis of the SafeBoosc II trial (ReviewPLOSone-2020-1005)
This report is an exploratory post-hoc analysis of the SafeBoosC II trial to assess if there is a relationship between the burden of cerebral hypoxia and neurodevelopmental outcome at 2 years corrected age. The SafeBoosC II trial was a randomized study among 166 preterm infants to determine if continuous NIRS recording of cerebral oxygenation over the first 72 hours after birth combined with a protocol to maintain cerebral oxygenation between 55-85% reduces the time with cerebral hypoxia or hyperoxia. A series of publications have demonstrated that the intervention could reduce the time with cerebral hypoxia but outcome measures (biomarkers, EEG, imaging, neurodevelopment) have not be altered. The current manuscript builds on an earlier publication reporting that infants in the highest quartile of cerebral hypoxia exposure had more severe intracranial hemorrhage, altered EEG or death compared to infants in the lowest 3 quartiles.

Comments:

1) Methods: Although there have been multiple publications from this trial which provide details of the study, it would be helpful for readers not familiar with the trial to provide a brief description of the study within the methods section (more than currently provided).

We tried to keep the method section short – as the study has all ready been described in detail elsewhere. However it might have been reduced too much. We have added more information in the method section, and believe that it has improved.

2) Methods: Since the results for the CUS are reported, there should be a description of mild, moderate and severe brain injury.

The following description has been added:

*No brain injury: None of the findings below.*

*Mild/moderate brain injury: Grade 1-2 IVH (including germinal layer haemorrhage), Isolated ventriculomegaly with ventricular index <p97a, Inhomogeneous flaring persisting after day 7, or Global thinning of corpus callosum at TEA.*

*Severe brain injury: Intra ventricular haemorrhage grade III (ventricular index >p97 during the acute phase), Post haemorrhagic ventricular dilatation, Parenchymal/periventricular haemorrhagic infarction, Unilateral porencephalic cysts, Cystic periventricular leukomalacia (bilateral), Cerebellar haemorrhage, Cerebral atrophy at term age or Stroke.*

3) Methods/neurodevelopmental assessments: Were examiners blinded to treatment intervention and other clinical data such as imaging results?

Following has been added in the method section.

*The parents filling out the ASQ questionnaires were not blinded to intervention nor imaging results.*

*The psychologists conducting the tests were blinded to cerebral NIRS outcome and imaging results.*

4) Methods: definition of neurodevelopmental impairment and CP belong in the methods and not the legend of a table.

The section has been moved from legend to Table 1 to the method section in the main manuscript,

5) Methods/statistics: The sentence "If baseline patient characteristics, the interventions conducted during the 72 hours of NIRS... is a run on sentence and is awkward. Although it is recognized that there was no adjustment for neonatal complications, it is not clear why one would consider doing any adjustment for neonatal complications since they may be in the causal path to the outcome at 2 years.

In the reviewed manuscript we write:

If baseline patient characteristics, the interventions conducted during the 72 hours NIRS monitoring, or the neonatal complications rate differed significantly and the differences between the developmental outcomes in the two groups, an additional multiple logistic regression analysis was planned – to eliminate the effect of confounding.

The sentence has been changed into:

If baseline patient characteristics, the interventions conducted during the 72 hours NIRS monitoring, or the neonatal *morbidity rate (severe brain injury on cUS, necrotising enterocolitis, broncopulmonary dysplasia or retinopathy of prematurity)* differed significantly (p<0.05) and the differences between the developmental outcomes in the two groups were significant (p<0.05), additional multiple logistic and linear regression analyses was planned *– as the basis for discussing the possibilities as regards causal paths as well as confounding. By request of the reviewers, these multiple regression analyses as well as regression analyses using the cerebral hypoxia as a continuous independent variable were done for the purpose of exploring the sensitivity/stability of the main results.*

6) Methods/statistics: The two most important issues are as follows;

a. Death is not accounted for in the analyses. Shouldn't the primary outcome be death or a measure of neurodevelopment and a secondary outcome of neurodevelopment among survivors as presented? A value below the lowest measured values for cognitive and motor functions could be assigned to allow an assessment of death for this analysis.

We already reported on the effect of cerebral hypoxia on the risk of death (Plomgaard et al 2017). In this manuscript our interest is to investigate cerebral hypoxia as a predictor/cause of NDI in extremely preterm infants. While others may have planned statistics otherwise, we would be uneasy about changing the analysis now, when we took care in defining the statistical analysis plan a priory.

b. There is no adjustment for baseline differences in characteristics among the lowest 3 quartiles and the highest quartile of infants (eg, p values < 0.2 or some other cut point for univariate analyses).

We choose a priory to adjust if the p-value was below 0.05. This did not happen

7) Results: the list of baseline characteristics is quite short. Is this the extent of data that is available? Is there information regarding maternal health (diabetes, hypertension), maternal education or sociodemographic status, placental problems (abruption, previa), mode of delivery, histological chorioamnionitis, intubation at birth?

Thank you for this comment.

We do not have any data on socio-economic status and collected very limited data on pregnancy, delivery, and neonatal care. SafeBoosC-II was an investigator-sponsored multicentre randomised trial with a limited budget, and one of the trade-offs was to do with limited dataset. The randomised design should overcome some of this limitation.

We did not collect data on histological chorioamnionitis only information about clinical chorioamnionitis - this is included in table 1.

Intubation at birth was not registered – but mechanical ventilation during the first 72 hours is included in table 1.

8) Results: umbilical pH is not a treatment.

Umbilical pH has been moved to “patient characteristics” in table 1

9) Results/treatment in the first 72 hours; is their information on pneumothorax and placement of a chest tube?

We have no information on pneumothorax or placement of chest tube. We only have data on mechanical ventilation. This is included in table 1.

10) Results: the statement "neonatal complication rates were similar" needs more detail as to what complications are being referred to.

This statement refers to table 1, where following complications are listed: severe brain injury on cUS, Necrotising enteroclitis, broncopulmonary dysplasia and retinopathy of prematurity.

We have changed our wording “neonatal complications” into “major neonatal morbidities” “major neonatal morbidities”.

“***Table 1***” has been added.

11) Results: "The rates of developmental impairment tended to be increased..." Reporting of trends can be problematic and sometimes misleading. It may be more objective to state that the point estimate for outcome x was in the direction of an increase in impairment but the confidence limits were very wide.

We agree that the confidence intervals are wide and we have now added an effect size:; (Cohen’s d = -0.40, 95% CI: -0.12 to 0.93, p = 0.13).

12) Discussion/1st paragraph: Discussing trends can be problematic. Consider a more objective statement that the wide confidence limits precludes the ability to assert that there is or is not an association between 2 year outcome and cerebral hypoxia.

We have deleted following from the discussion section:

However, there was a trend towards more severe developmental impairment in the high burden group, but the confidence limits of the odds ratio were wide.

13) Discussion: the statement "The fact that the difference in the cerebral outcomes waned with age underline the multifactorial influence in long term outcome..." is an important statement and could be viewed as the conclusion. The authors need to do a better job of integrating this possible conclusion with the limitation of their data. For example as the authors note, although the difference in Bayley scores is approximately a third of a standard deviation in favour of less cerebral hypoxia, the confidence limits for neurodevelopmental impairment are wide. This study is relatively small for 2 year outcome and as noted in the limitations may be under powered. Better integration of these comments would help the message. It seems like it is an open question.

Thank you for this comment. We tried to do better, and have now stated:

We were not able to demonstrate a similar association to neurodevelopmental deficits at two years of age. This may just be due to the limited statistical power. The confidence limits included clinical relevant associations. Also, it is possible that the ‘true’ effects of cerebral hypoxia ‘wane’ with age due to the multifactorial aetiology of long term outcome in preterm infants. In particular, the relation is not perfect between severe brain injury as defined by cranial ultrasound imaging in the neonatal period and moderate-to-severe neurodevelopmental deficit at two-years or later.

14) Discussion: not performing adjustment of baseline characteristics because there were no differences in the outcome seems very unconventional.

We were keen to avoid statistical over-use of this dataset – in particular keen to limit the ‘positivity bias’. But, given the concerns of both editors, we have now done an adjusted analysis to judge the sensitivity/stability of the main results.

This is illustrated in figure 2.

15) Conclusion: This statement should reflect that this was an exploratory analysis. In addition stating that cerebral hypoxia was not statistically associated with 2 year outcome may be misconstrued as more definitive than it is. The wide confidence limits limit the ability to draw conclusions and further study is needed seems like a better way to summarize this interesting data.

Following has been added in the discussion section:

We were not able to demonstrate a similar association to neurodevelopmental deficits at two years of age. This may just be due to the limited statistical power. The confidence limits included clinical relevant associations. Also, it is possible that the ‘true’ effects of cerebral hypoxia ‘wane’ with age due to the multifactorial etiology of long term outcome in preterm infants.

And we have changed a sentence in the conclusion section and added *“but the confidence limits were wide”:*

The burden of cerebral tissue hypoxia was not statistically significantly associated with 2-year neurodevelopmental outcome in this post-hoc analysis of data from a randomised controlled trial, *but the confidence limits were wide*. A larger, appropriately powered randomised trial is needed to test the clinical value of cerebral oximetry in preterm infants.

Reviewer #2: -

This manuscript should include a flow diagram showing how the children recruited to the overarching trial whittle down to the number included in this analysis.

A flowchart is now included in the manuscript as Figure 1.

*Title: Flowchart from inclusion to 2-year neurodevelopmental outcome*

Figure1 in the original manuscript has been renamed into figure 2.

- In the methods, a description of the various tools used for neurodevelopmental evaluation should be included.

Following has been added to the method section.

*Medical examination: Basic growth measurements were collected. Vision and auditory functions were evaluated. If the child showed signs of cerebral palsy, the gross motor function was assessed using the Gross Motor Function Classiﬁcation System (GMFCS). The doctor performing the medical examination was not blinded to the intervention.*

*Bayley Scales of Infant and Toddler Development, Second Edition (Bayley- II) or Third Edition (Bayley-III), depending on what version the centre was using at the time of the study. …….*

*The psychologists conducting the tests were blinded to cerebral NIRS outcome and imaging results.*

*ASQ: A set of parental questionnaires covering the development of children aged from four months to ﬁve years. Each questionnaire contains ﬁve developmental domains, communications, gross motor skills, ﬁne motor skills, problem solving and personal–social skills. The parents returned the questionnaires by mail or handed them in on the day of medical examination. The parents were not blinded to intervention group nor imaging results.*

- The statistical analysis requires a number of improvements
(1) the table of descriptive characteristics of should be separated from the table describing associations between the main exposure and outcomes. Descriptive characteristics should be summarised in terms of counts and percentages for categorical ones and either means and SDs or medians and IQRs or ranges, for continuous ones. Statistical tests comparing groups are not necessary at this stage, unless the authors have some a-priori hypotheses to test - otherwise these tests simply constitute data dredging/multiple testing (which would be difficult to adjust for in this underpowered study).

Thank you for carefully reviewing our table.

We have split Table 1 into three smaller tables.

**Table 1**

Now only contains patient characteristics and no p-values.

**Table 2**

Consists o the interventions during the first 72 hours and the major neonatal morbidities at term.

**Table 3**

Only includes the 2-year follow-up results.

(2) a separate table presenting the associations between the main exposure and the various outcomes should be presented. For each outcome either counts (%) or means (SEs) should be presented for each group, followed by the effect estimate, its 95% confidence intervals and p-values. p-values should not be included for subgroup analyses, e.g. the various components of the neurodevelopmental outcome scores, as this also constitutes multiple testing.

Odds ratios has been added to table 3

Although the authors have previously described the main exposure by contrasting the lower three quartiles of burden of cerebral hypoxia to the uppermost, this approach collapses potentially heterogeneous individuals into fewer groups resulting in loss of information - this is particularly critical in this study where there are few individuals and a small number of outcome events. The authors should include sensitivity analyses where the main exposure is treated as a continuous outcome, to explore linear (or log-linear) trends and departures from linearity/log-linearity in the association between burden of hypoxia and each of the outcomes.

We have done that and added to the methods section that we did it on the reviewers’ request. The results are presented in a compact form – in figure 2.

Additionally, more careful consideration should be made about how potential confounders are identified and controlled for. Only potential confounders which could plausibly herald hypoxia (and are not on a causal pathway in the association between the main exposure and each outcome) should be further explored for adjustment.

Thank you for this comment. Since our statistical analysis did not bring us to the adjusted analysis, we have tried to address these important issues in the discussion section.

The methods, results and discussion should then be updated to reflect these changes.

6. PLOS authors have the option to publish the peer review history of their article ([what does this mean?](https://journals.plos.org/plosone/s/editorial-and-peer-review-process#loc-peer-review-history)). If published, this will include your full peer review and any attached files.


If you choose “no”, your identity will remain anonymous but your review may still be made public.


**Do you want your identity to be public for this peer review?** For information about this choice, including consent withdrawal, please see our [Privacy Policy](https://www.plos.org/privacy-policy).

Reviewer #1: No

Reviewer #2: No

[NOTE: If reviewer comments were submitted as an attachment file, they will be attached to this email and accessible via the submission site. Please log into your account, locate the manuscript record, and check for the action link "View Attachments". If this link does not appear, there are no attachment files.]

While revising your submission, please upload your figure files to the Preflight Analysis and Conversion Engine (PACE) digital diagnostic tool, [https://pacev2.apexcovantage.com/](https://pacev2.apexcovantage.com/). PACE helps ensure that figures meet PLOS requirements. To use PACE, you must first register as a user. Registration is free. Then, login and navigate to the UPLOAD tab, where you will find detailed instructions on how to use the tool. If you encounter any issues or have any questions when using PACE, please email PLOS at [figures@plos.org](mailto:figures@plos.org). Please note that Supporting Information files do not need this step.
