# Supplementary material for: Early cerebral hypoxia in extremely preterm infants and neurodevelopmental impairment at 2 year of age: A post hoc analysis of the SafeBoosC II trial
Source: PLoS One. 2022 Jan 24;17(1):e0262640. doi: 10.1371/journal.pone.0262640 (PMC8786171; doi:10.1371/journal.pone.0262640)
Supplement: Attachment — Submitted filename: response_to_reviewers.docx [file pone.0262640.s002.docx]

Point-by-point response to reviewer 2 (in italics):

I find this manuscript a bit more confusing since the last round of reviews. My understanding is that the authors are exploring the association between cerebral hypoxia (the main exposure) and 2-year outcomes including neurodevelopmental scores, growth, vision and auditory functions, cerebral palsy, etc.
The reason for my confusion is that the approach to the analysis is not clearly articulated and seems incorrect in various places, therefore I am unable to make any sense of the results.

*Sorry. But yes, this is our aim. We have expanded the last sentence in the introduction section, and included a sentence on the goal in the section on statistics*

In particular, it is confusing why comparisons such as that in Table 2 are presented: if the hypothesis is that burden of hypoxia might affect outcomes, then performing a statistical test to check whether there are differences in neonatal morbidities and treatments between the groups does not serve any purpose, especially when those morbidities and treatments could influence the outcomes of interest here i.e. these morbidities are on the causal pathway between the main exposure and the outcome. I feel that the authors have not clearly considered the potential relationships (and the directions of those potential relationships) between the factors explored, therefore some of the analyses conducted don't really serve a useful purpose.

*We disagree that statistical tests of the difference in neonatal variables between the two hypoxic burden groups are irrelevant. We think that they may help the reader judge whether the differences present in this data set may represent ‘true’ associations with neonatal brain hypoxia. But we completely agree with the reviewer that the challenge of causal inference in the context of multiple, partly simultaneous measures of pathophysiology is massive. Therefore, we a priory decided not to embark on an attempt of multivariable analysis if the association between hypoxic burden and neurodevelopmental outcomes were statistically insignificant – which was the case. We have made our position clearer in the section on statistics and in the discussion section.*

Another bit of analysis that doesn't make sense is in Table 3. For example, odds ratios are presented for mental development index and ASQ scores - these are continuous outcome measures for which I would have expected to see mean difference in these measures between the two cerebral hypoxia groups. Presenting odds ratios implies that the authors have explored the effects of the neurodevelopment scores as exposures on hypoxia as the outcome - which is clearly the wrong direction of potential causal relationship between the factors, since the hypoxia was assessed 15 minutes after birth and the nourodevelopmental outcomes at 2 years; therefore the hypoxia is the exposure and the neurodevelopmental score the outcome in this relationship.

*We apologize for severe problems with table 3. The correct mean differences and OR with 95% confidence limits are now presented*

Here is what I would suggest the authors do to focus this analysis more clearly:
- make very clear in the methods what the main exposure and outcome are

*We have done that*

- the table of descriptive characteristics should present the participant characteristics (already present in table 1), treatments received, and morbidities in the two exposure groups. There is no need to conduct statistical tests comparing these groups in terms of these factors unless some factors are identified which are associated with the main exposure and the main outcomes and not on the causal pathway between them - the problem with the current analysis is that some of these factors are potentially on the causal pathway (for example, the burden of cerebral hypoxia could influence treatment received or neontatal morbidity which could then influence outcomes). Being on the causal pathway means even if the factor is associated with exposure, it is inappropriate to consider adjusting for it in the analysis of the outcome.

*We share the reviewer’s views on the need of considering statistical associations as well as pathophysiological concepts defining likely causal pathways, including variables that may best be considered as mediators, before embarking on attempts of adjusting for confounding relations. In this paper, however, due to the small sample size as well as the complex pathophysiology and the lack of association between hypoxic burden and neurodevelopmental outcome, we have given up on this and have focused on presenting data that potentially may be used in metaanalyses. Regarding causality, it is our opinion that this question requires a trial of intervention for an answer.*

- the table of outcomes should list the main outcomes, the means and standard errors of the continuous ones or the counts and proportions/percentages of the binary/categorical ones in each of the hypoxia groups, followed by the estimate of association e.g. mean difference, 95%CI and p-value for continuous outcomes and ORs or RRs with 95%CI and p-values (in that order) for binary outcomes. This analysis should be limited to the main outcomes only and not their components as this would result in multiple testing which there isn't enough power in this study to adjust for.

*Thank you. We have done that.*


Minor comments

- the statement just above the 'statistics' section relating to figure 1 belongs to the results section.

*Moved*

- the denominators for each column in Table 2 are missing; there's only 'n=' at the top of each column.

*We apologize. They are now included*

- as advised previously, please report the standard errors (SEs) not standard deviations (SDs) for continuous outcomes in Table 3. SEs are preferred for inference while SDs are appropriately used for description in Table 1.

*We agree in principle, but the p-values also serve to support the interpretation as regards inference, and it is more conventional to give SDs to serve the interpretation of differences on a population scale. So we have not changed to SE*

- also, please report the p-value after the effect estimate and its 95%CI in Table 3.

*Done*

- the statistical methods say you compared the baseline patient characteristics; in fact, you presented the baseline characteristics, which is appropriate to do i.e. no comparison required, and then you compared the interventions, complications and outcomes between the two groups. Please clarify these in the methods.

*We hope that this is clear now.*
