# Supplementary material for: Early cerebral hypoxia in extremely preterm infants and neurodevelopmental impairment at 2 year of age: A post hoc analysis of the SafeBoosC II trial
Source: PLoS One. 2022 Jan 24;17(1):e0262640. doi: 10.1371/journal.pone.0262640 (PMC8786171; doi:10.1371/journal.pone.0262640)
Supplement: Attachment — Submitted filename: responce to reviewer.docx [file pone.0262640.s003.docx]

**Comments to the Author**

Review Comments to the Author

Please use the space provided to explain your answers to the questions above. You may also include additional comments for the author, including concerns about dual publication, research ethics, or publication ethics. (Please upload your review as an attachment if it exceeds 20,000 characters)

Reviewer #2: - the authors' response regarding the reporting of SDs instead of SEs in Table 3 is unsatisfactory. The authors say that "it is more conventional to give SDs to serve the interpretation of differences on a population scale" - this is incorrect. SDs are descriptive of a sample only. The equivalent statistic that applies to the population is the SE. Please see Altman & Bland's brief discussion of this at [https://www.ncbi.nlm.nih.gov/pmc/articles/PMC1255808/](https://www.ncbi.nlm.nih.gov/pmc/articles/PMC1255808/). Furthermore, their response that "the p-values also serve to support the interpretation as regards inference" is only partly correct, as p-values are used to make inference with regards to the null hypothesis only; on their own they do not give any information on the uncertainty about estimates of effect or association, which the SEs provide. It remains my recommendation that the SDs in Table 3 and in the text pertaining to the outcomes should be replaced with SEs as is the standard practice in reporting estimates of effect or association for inference.

*We thank the reviewer for patient teaching. We agree completely with the points made by Altman & Bland as regards the need to avoid confusion, but – perhaps due to our own habits – find it easy to use the information on sample size and SDs to do gross estimations of SEs, if they are not reported. To finalise our manuscript, however, we have followed the recommendations of the reviewer, and hope that the result is satisfactory.*

- all tables need one more improvement: wherever proportions are reported (e.g. proportion of male sex in table 1), these need to be accompanied by counts i.e. in this example, the number of males. Similarly wherever counts are reported e.g. in table 3, the counts for cerebral palsy, these need to be accompanied by the proportions.

*Upon request we have added the proportions in table 1 and 2 and the counts in 3.*

- there is still need for some improvement in how the study is described; for example, in the first paragraph on page 4, I needed several reads to understand that the sentence that begins with 'Control group,' was a section describing the control group.

*We have added a colon in the text – and hope this has made the sentence clearer.*

*Control group: the cerebral oxygenation levels were recorded, but the clinicians were blinded to the results and the infants were given standard treatment and care, only.*

- aspects of the reporting of results also still need improvement; wherever comparing differences between the hypoxia groups, this needs to be clear. For example, statements such as "there were no differences between the Bayley... scores" in the abstract and results sections need to be explicit that this comparison was between the hypoxia groups.

*We did all ready describe the two groups of patients according to the burden of cerebral of hypoxia in the method section. We have made a few modifications in the abstract and the result section according to this comment and believe the text has been improved.*
